# The relationship between MRI-detected hip abnormalities and hip pain in hip osteoarthritis: a systematic review

**DOI:** 10.1007/s00296-024-05678-2

**Published:** 2024-08-13

**Authors:** Haonan Fang, Xiaoyue Zhang, Junjie Wang, Xing Xing, Ziyuan Shen, Guoqi Cai

**Affiliations:** 1 Department of Epidemiology and Biostatistics, School of Public Health, Anhui Medical University, Hefei, 230032 Anhui China; 2 Menzies Institute for Medical Research, University of Tasmania, Hobart, TAS 7000 Australia

**Keywords:** Hip joint, Hip osteoarthritis, Hip pain, Magnetic resonance imaging, Pain

## Abstract

Magnetic resonance imaging (MRI) is increasingly used in the classification and evaluation of osteoarthritis (OA). Many studies have focused on knee OA, investigating the association between MRI-detected knee structural abnormalities and knee pain. Hip OA differs from knee OA in many aspects, but little is known about the role of hip structural abnormalities in hip pain. This study aimed to systematically evaluate the association of hip abnormalities on MRI, such as cartilage defects, bone marrow lesions (BMLs), osteophytes, paralabral cysts, effusion-synovitis, and subchondral cysts, with hip pain. We searched electronic databases from inception to February 2024, to identify publications that reported data on the association between MRI features in the hip joint and hip pain. The quality of the included studies was scored using the Newcastle-Ottawa Scale (NOS). The levels of evidence were evaluated according to the Cochrane Back Review Group Method Guidelines and classified into five levels: strong, moderate, limited, conflicting, and no evidence. A total of nine studies were included, comprising five cohort studies, three cross-sectional studies, and one case-control study. Moderate level of evidence suggested a positive association of the presence and change of BMLs with the severity and progress of hip pain, and evidence for the associations between other MRI features and hip pain were limited or even conflicting. Only a few studies with small to modest sample sizes evaluated the association between hip structural changes on MRI and hip pain. BMLs may contribute to the severity and progression of hip pain. Further studies are warranted to uncover the role of hip MRI abnormalities in hip pain. The protocol for the systematic review was registered with PROSPERO (https://www.crd.york.ac.uk/PROSPERO/, CRD42023401233).

**Supplementary Information:**

The online version contains supplementary material available at 10.1007/s00296-024-05678-2.

## Intruduction

Osteoarthritis (OA) is a common musculoskeletal disease of the entire joint, characterized by pain and disability [1]. The hip joint is a frequently affected site of OA [2], affecting more than 240 million people in the world [3]. The pathophysiology of OA involves multiple tissues, including cartilage, bone, ligaments, synovium, and muscles [2, 4]. Understanding the involvement of these tissues in joint symptoms is crucial for developing effective treatment strategies. Although conventional x-rays are frequently used for the diagnosis and classification of OA, soft tissues cannot be adequately evaluated using this technique. Moreover, the available evidence does not show a consistent association between radiographic features and OA pain [5, 6]. More advanced imaging techniques, especially magnetic resonance imaging (MRI), offer much higher sensitivity in detecting early signs of joint damage, making it an invaluable tool for evaluating OA and its associated pain [7, 8].

In contrast to the extensive body of research examining factors associated with knee pain, there has been much fewer studies investigating the source of hip pain [9]. The xcharacteristics of hip OA differ significantly from knee OA in many aspects including epidemiology, prognosis, pathophysiology, anatomical and biomechanical factors, clinical presentation, and pain management [10]. Thus, the etiology and contributing factors for hip pain can differ from those of knee pain. It has been shown that knee pain is associated with several MRI features such as bone marrow lesions (BMLs) [11], effusion/synovitis [12, 13], meniscus tear, infrapatellar fat pad [14], osteophytes [15] and cartilage defects [16]. Clinical studies have gone further to explore the use of BMLs and effusion-synovitis as treatment targets for knee OA [17–20]. However, few studies have evaluated the role of MRI features in the hip in the assessment, prognosis, and treatment of hip OA. Therefore, this study aimed to systematically review studies evaluating the association between MRI abnormalities and hip pain.

## Materials and methods

### Protocol registration

The protocol for the systematic review was registered with PROSPERO (https://www.crd.york.ac.uk/PROSPERO/, CRD42023401233). This systematic review was reported according to the Preferred Reporting Items for Systematic Reviews and Meta-Analyses (PRISMA) checklist [21]. The report of this study followed the Cochrane Handbook for Systematic Reviews of Interventions [22]. This study was a systematic review and ethics committee review was not applicable.

### Data source and search strategy

We searched Medline (via Ovid), Web of science, Embase (via Ovid), and Cumulative Index to Nursing & Allied Health Literature (CINAHL) from inception to June 2024, for relevant studies evaluating the association of MRI abnormalities in the hip with hip pain. The following search terms were used: ‘hip’, ‘hip joint’, ‘pain’, ‘MRI’, ‘osteoarthritis’, detailed search strategies are provided in the Supplementary Methods. We also checked the citation lists of the included studies and relevant systematic reviews and gray literature (e.g. conference abstract) for additional studies.

### Study selection

Two authors (HF and XZ) conducted an independent review of the titles and abstracts of all identified studies, followed by retrieving the full texts of relevant studies for further screening. The full-text reviews were performed in accordance with the selection criteria outlined in the registered protocol. Specifically, observational studies evaluating the association between MRI abnormalities (e.g. BMLs or cartilage defect) and pain in the hip joint were included. Animal studies or studies without data on MRI features and/or hip pain were excluded. There was no restriction on language.

### Data extraction

Two authors (HF and XZ) independently extracted data from each included study. The extracted data included: (1) study characteristics (the first author, year of publication, place (country/territory), study design, and sample size); (2) characteristics of the study population (e.g. age, sex, OA patients or community-dwelling participants); (3) MRI features (e.g. subchondral cysts, paralabral cysts, cartilage defects, BMLs, osteophytes, and effusion/synovitis) (Table 1); (4) assessment of hip pain, (5) main findings for the association between MRI features and hip pain; and (6) adjusted covariates.

**Table 1:** Definitions of MRI structural abnormalities

Structural abnormalities	Definitions
Subchondral cysts	A rounded area under the cartilage filled with fluid
Paralabral cysts	Small and common fluid lesions that are closely associated with acetabular labral tears
Cartilage defects	A focal area of damage to the articular cartilage
Bone marrow lesion	Areas of increased signal intensity adjacent to the subchondral bone
Osteophytes	Localized bony outgrowths that extended from bones cortical surface
Effusion-synovitis	An irritated and inflamed synovial membrane, resulting in an imbalance in fluid secretion and the formation of a fluid buildup

### Assessment of study quality and credibility of evidence

Two authors (HF and XZ) independently assessed the methodological quality of the included studies using the Newcastle-Ottawa Scale (NOS) for cohort studies [23] and case-control studies [24], and an extension for cross-sectional studies [25]. Differences in scoring were resolved by discussion or by consulting the third author (GC). The possible scores of study quality ranged from 0 to 9 for cohort studies, 0–8 for cross-sectional and case-control studies, with higher scores indicating higher quality. A score of ≥ 7 was considered high study quality for cohort studies [26], cross-sectional studies [27] and case-control studies [28].

The same two authors independently evaluated the credibility of evidence for the association between each MRI feature and hip pain on the basis of the guidelines of the Cochrane Collaboration Back Review Group [29]. The credibility of evidence was categorized into five levels based on the following criteria: (1) Strong: multiple high-quality cohort studies show generally consistent findings, (2) Moderate: One high-quality cohort study and at least two high-quality cross-sectional studies or only at least three high-quality cross-sectional studies show generally consistent findings, (3) Limited: a single cohort study, or up to two cross-sectional studies show less consistent findings, (4) Conflicting: no consistent findings were reported, (5) No evidence: no studies were published.

## Result

### Literature search

**Fig. 1:**
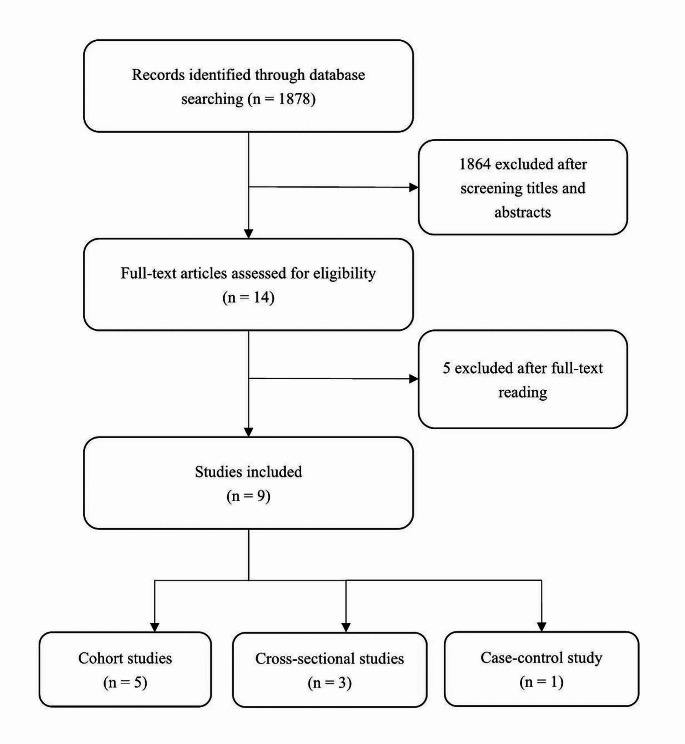
Flowchart of study selection

The flowchart of the study selection process is shown in Fig. 1. We identified a total of 1878 potentially relevant records from electronic search. After screening the titles and abstracts, 1864 were excluded. From the remaining 14 records, we further excluded 5 irrelevant studies, leaving 9 studies in this systematic review. Among the studies included, 5 were cohort studies [30–34], 3 were cross-sectional studies [35–37], and 1 was a case-control study [38].

### Characteristics of included studies

Table 2 shows the characteristics of included studies. Overall, the sample size of the studies were small to modest (*n* = 19 to 237), and the follow-up time of the 5 cohort studies ranged from 1 to 2.3 years. Among the 9 included studies, 4 examined multiple MRI features [32, 35, 37, 38] and 5 examined a single MRI feature [30, 31, 33, 34, 36]. Three studies were conducted in the same population [30, 33, 34]. Four studies used a 1.5T MRI [30, 33, 34, 36], four used a 3T scanners [31, 32, 35, 38], and the remaining one did not report the strength of MRI used [37]. Most of the studies used sagittal imaging [30, 32–34, 37, 38], with two studies using both sagittal, coronal and oblique axial imaging [32, 38], only one study used coronal and sagittal imaging [35], and one study used coronal imaging alone [36]. The patients investigated in the included studies were essentially middle-aged and older adults (mean age 46.5 to 66 years, 27.6-57.9% males), except for one study that examined high-impact athletes in their 20s and 30s [38].

**Table 2:** Characteristics of included studies

	Study design	Population	MRI features	Pain assessment
Ahedi 2014[30]	Cohort, 2.3 years	198 community-dwelling adults in Australia (56% male; age 64.4 ± 6.9 years)	BMLs	WOMAC pain ^a^
Koyama 2022[31]	Cohort, 1 year	84 patients with anterior hip pain and catching in Japan (36.9% male; age 46.5 ± 1.6)	BMLs	MHHS pain ^a^
Schwaiger 2016[32]	Cohort,1.5 years	54 community-dwelling adults in the US (57.4% male; age 47.2 ± 13.2 years)	BMLs, subchondral cysts, paralabral cysts	HOOS pain ^b^
Ahedi 2016[33]	Cohort, 2.3 years	194 community-dwelling adults in Australia (age 64.7 ± 7.2 years)	Cartilage defects	WOMAC pain ^a^
Ahedi 2020[34]	Cohort, 2.3 years	196 community-dwelling adults in Australia (44.4% male; age 64.2 ± 4.9 years)	Effusion-synovitis	WOMAC pain ^a^
Kijima 2020[37]	Cross-sectional	84 osteoarthritis patients undergoing surgery in Japan (27.6% male; mean age 63 years, range 42–83)	Cartilage defects, BMLs, paralabral cysts, osteophytes, effusion-synovitis	VAS pain ^a^
Kumar 2013[35]	Cross-sectional	85 community-dwelling adults in the US (51.8% male; mean age 47 ± 3.57 years)	Cartilage defects, BML, subchondral cysts	HOOS pain ^b^
Taljanovic 2008[36]	Cross-sectional	19 patients undergoing total hip arthroplasty in the US (57.9% male; mean age 66 years)	BMLs	The D’Aubigne-Postel grading ^b^
Heerey 2021[38]	Case-control	237 high-impact athletes (78.8% male; mean age 26 years, range 23–31 years)	BMLs, subchondral cysts, effusion-synovitis, cartilage defects	Presence/absence

HOOS, Hip disfunction and Osteoarthritis Outcome Score; MHHS, Modified Harris Hip Score; VAS, Visual Analogue Scale; WOMAC, Western Ontario and McMaster Universities Arthritis Index

a Higher scores indicate more extreme symptoms

b Lower scores indicate more extreme symptoms. To allow direct comparison, the correlation coefficients between MRI abnormalities and hip pain were transformed so that positive values denote positive correlations

### Assessment of study quality

Five of the 9 studies (55.6%) were scored above the high-quality threshold (i.e. ≥7) according to the NOS assessment. For cross-sectional studies, only 1 of the 3 studies was scored high-quality, with the main issues being small sample size, sample representativeness, and the lack of comparison between respondents and non-respondents. Meanwhile, 4 of the 5 cohort studies were above the high-quality threshold, and only 1 study had issues with the representation and selection of exposed and non-exposed groups (Supplementary Tables 1–3).

### Association between MRI features and hip pain

### Subchondral cysts

One cross-sectional study [35], one case-control study [38] and one cohort study [32] evaluated the association between subchondral cysts and hip pain (Table 3). The credibility of the evidence was limited. The cross-sectional study showed a positive correlation between total subchondral cyst score (grade 0–2) and more severe hip pain score, assessed by the Harris Hip Score and Hip Disability and Osteoarthritis Outcome Score (HOOS) pain subscale (rank correlation coefficient = 0.37, *P* = 0.001) [35]. The case-control study did not observe a significant difference in subchondral cysts (grade 0–2) between symptomatic and control hips in athletes (8% vs. 7%, odds ratio (OR) = 1.29, 95% confidence interval (CI) 0.51 to 3.23) [38]. The cohort study showed a boardline significant association between baseline subchondral cyst score (grade 0–2) and change in hip pain (rank correlation coefficient = 0.30, *p* = 0.051) [32]. Moreover, the cohort study found a significant correlation between progression of subchondral cysts and change in HOOS symptoms other than pain (i.e. functional disability and stiffness) (rank correlation coefficient = 0.30, *p* = 0.03) but not hip pain score over 1.5 years (rank correlation coefficient = 0.18, *p* = 0.19) [32].

**Table 3:** Summary of the associations between MRI abnormalities and hip pain

Studies	Study design	Association *		Adjusted confounders	Number of studies: positive/total (%)	
		Crude	Adjusted		All	High quality
Subchondral cysts						
Schwaiger, 2016[32]	C	Baseline subchondral cysts and pain: r_s_ =0.30, *p* = 0.051 Δ subchondral cysts and Δ pain: r_s_ =0.182, *p* = 0.188	-	NA	1/3 (33.3%)	0/0
Kumar, 2013[35]	CS	r_s_ =0.37, *p* = 0.001	-	NA		
Heerey, 2021[38]	CC	Men: OR (95%CI): 1.12 (0.45, 2.76)	Men: OR (95%CI): 1.29 (0.51, 3.23)	age, BMI		
Paralabral cysts						
Schwaiger 2016[32]	C	Δparalabral cyst and Δ pain: r_s_ =0.155, *p* = 0.262 ΔParalabral cyst and Δ pain: r_s_ =0.155, *p* = 0.262	-	age, sex, BMI, and KL	0/2	0/0
Kijima, 2020[37]	CS	*r* = 0.6546, *p* < 0.0001; mean paralabral cyst score: 0.91 vs. 0.81 for painful and non-painful hip, *p* = 0.3908	-	NA		
Effusion-synovitis						
Ahedi, 2020[34]	C	-	Presence of hip effusion-synovitis at two/three sites and presence of hip pain: PR (95% CI): 1.42 (1.05, 1.93)	age, sex, BMI	2/3 (66.6%), conflicting direction	1 (C) /2 (1 C, 1CS) (50%)
Kijima, 2020[37]	CS	r_s_=0.3259, *p* = 0.161	-	NA		
Heerey, 2021[38]	CC	OR (95%CI): 0.46 (0.26, 0.81)	OR (95%CI): 0.46 (0.26, 0.81)	age, sex, BMI		
Cartilage defects						
Kijima, 2020[37]	CS	*r* = 0.4565, *p* = 0.0005	-	NA	3/4 (75%)	2 (1 C, 1CS) /3 (1 C, 2CS) (66.7%)
Kumar, 2013[35]	CS	r_s_=0.17, *p* = 0.146 (femoral cartilage) r_s_=0.25, *p* = 0.026 (acetabular cartilage)	-	NA		
Heerey, 2021[38]	CC	OR (95% CI): 1.13 (0.67, 1.91)	OR (95%CI): 1.13 (0.67, 1.91)	age, sex, BMI		
Ahedi, 2016[33]	C	Any cartilage defects and category 2 hip pain: PR (95% CI): 1.20 (1.02,1.35) Grade 2 cartilage defects and hip pain: PR (95% CI): 1.40 (1.09,1.80)				
Osteophytes						
Kijima, 2020[37]	CS	*r* = 0.5811, *p* < 0.0001	-	NA	1	0
BMLs						
Ahedi, 2014[30]	C	The severity of hip pain and Δ acetabular BML size: β (95% CI): 4.18 (1.54, 6.88) Any BML size and Δ hip pain: β (95% CI): 0.85 (0.00, 1.71)	-	NA	5/7 (71.4%)	2 (C) /5 (3 C,2CS) (40%)
Koyama, 2022[31]	C	non-BML vs. BML ≤ 1 cm: *p* = 0.022; non-BML vs. BML > 1 cm: *p* < 0.001	-	NA		
Schwaiger, 2016[32]	C	Baseline BMLs and Δ pain: r_s_ =0.365, *p* = 0.007	β (95%CI): 0.690 (0.464,0.913); *p* = 0.018	age, sex, BMI, and KL		
Kijima, 2020[37]	CS	*r* = 0.3846, *p* < 0.0041	-	NA		
Kumar, 2013[35]	CS	r_s_=0.29, *p* = 0.01	-	NA		
Taljanovic, 2008[36]	CS	r_s_=0.51, *p* < 0.05	-	NA		
Heerey, 2021[38]	CC	IRR (95%CI): 1.72 (0.40, 7.44)	IRR (95%CI): 1.75 (0.42, 7.26)	age, sex, BMI		

BMI, body mass index; BML, bone marrow lesion; C, cohort study; CI, confidence interval; CS, cross-sectional study; CC, case-control study; IRR, incidence rate ratio; NA, not applicable; KL, Kellgren-Lawrence grade; OR, Odds ratio; PR, Prevalence ratio. r, Pearson correlation coefficient; rs, Spearman rank correlation coefficient; β, regression coefficient

*For studies that evaluated hip pain using HOOS or the D’Aubigne-Postel grading system (lower scores indicate more extreme pain), the associations between MRI abnormalities and hip pain were transformed so that positive correlation coefficients denote positive correlations

### Paralabral cyst

One cross-sectional study [37] and one cohort study [32] evaluated the association between paralabral cyst and hip pain (Table 3). The credibility of the evidence was limited. The cross-sectional study found that paralabral cyst scores, based on the Hip Osteoarthritis MRI Scoring System (HOAMS), were similar in painless and painful hips (mean paralabral cyst score: 0.81 vs. 0.91, *p* = 0.39) [37]. Consistently, the cohort study found that neither baseline nor progression of paralabral cysts was associated with change in HOOS pain or other subscales, except that progression of paralabral cysts was associated with HOOS activity of daily living subscale (rank correlation coefficient = 0.30, *p* = 0.03) [32].

### Effusion-synovitis

One cross-sectional study [37], one case-control study [38] and one cohort study [34] showed inconsistent findings for the association between hip effusion-synovitis and hip pain (Table 3). The credibility of the evidence was conflicting. The cohort study observed a significant positive correlation between presence of hip effusion-synovitis at two/three sites and presence of hip pain (PR (95% CI): 1.42 (1.05, 1.93)), although there was no significant correlation between change in effusion-synovitis size and change in hip pain [34]. By contrast, the case-control study showed an inverse correlation between effusion-synovitis and the presence of hip symptoms (OR (95% CI) 0.46 (0.26, 0.81)), before and after adjusting for age, sex, and BMI [38]. The remaining cross-sectional study reported no significant associations between joint effusion/synovitis and hip pain [37].

### Cartilage defects

One cohort study [33], one case-control study [38] and two cross-sectional studies [35, 37] examined the association between cartilage defects and hip pain (Table 3). The credibility of the evidence was limited. The cohort study reported higher levels of Western Ontario and McMaster Universities Arthritis Index (WOMAC) hip pain in individuals with any type of hip cartilage defects (PR (95% CI): 1.20 (1.02, 1.35)) and secondary cartilage defects (PR (95% CI): 1.40 (1.09, 1.80)) [33]. One cross-sectional study reported a significant linear correlation between cartilage defects score and Visual Analogue Scale (VAS) hip pain (*r* = 0.46, *P* < 0.001), although cartilage defects score was not statistically significantly different between individuals with and those without hip pain (mean cartilage defects score: 1.23 vs. 0.75, *p* = 0.18) [37], another cross-sectional study found a significant correlation between acetabular cartilage score and HOOS pain (*r* = 0.25, *p* = 0.026), but there’s no correlation between femoral cartilage score and HOOS pain (r_s_=0.17, *p* = 0.146) [35].

### Osteophytes

One cross-sectional study [37] examined the relationship between MRI-detected osteophytes and hip pain (Table 3), showing a positive correlation between osteophyte score and VAS pain (*r* = 0.5811, *p* < 0.0001), and there was a higher osteophyte score in the inferomedial compartment in individuals with hip pain than those without (3.0 vs. 2.0, *p* = 0.03) [37]. The credibility of evidence was limited.

### BMLs

Three cohort studies [30–32], three cross-sectional studies [35–37], and one case-control study [38] evaluated the association between BMLs and hip pain (Table 3). The credibility of evidence was moderate. All three cohort studies consistently reported a significant association between BMLs and hip pain, with one showing that change in BML size was significantly associated with change in hip pain (regression coefficient [β] (95% CI): 0.85 (0.00, 1.71)), and the severity of hip pain was associated with a per square centimeter increase in the size of acetabular BML (regression coefficient [β] (95% CI): 4.18 (1.54, 6.88)) [30]. The second cohort study found that Modified Harris Hip Score (MHHS) pain score was significantly lower in individuals with BMLs than those without, regardless of the size of BMLs (*p* < 0.05) [31], and the third cohort study indicated that baseline BML size was significantly associated with worsening of HOOS pain subscale (regression coefficient [β] (95% CI): 0.690 (0.464, 0.913)) [32]. All three cross-sectional studies reported positive correlations between BML scores and hip pain (*r* = 0.29 to 0.51, *p* < 0.05) [35–37], and the remaining case-control study did not observe a significant differences in BML scores between symptomatic and control hips [38].

## Discussion

This systematic review screened and evaluated studies that described the association between MRI-detected hip abnormalities and hip pain, and several MRI features were identified, such as osteophytes, subchondral cysts, paralabral cysts, effusion-synovitis, BMLs and cartilage defects. Overall, the number, sample size, and quality of included studies were inferior to studies focusing on the knee, and current evidence suggests that BMLs, cartilage defects, and osteophytes may be associated with the presence and severity of hip pain, while subchondral and paralabral cysts may not. Moreover, the association between effusion-synovitis and hip pain was conflicting. Considering the paucity of studies examining their association, a robust conclusion cannot be reached [39]. Thus, more studies are required to validate whether these MRI features contribute to the presence and severity of hip pain.

The credibility of evidence for the association between each of the hip MRI features and hip pain was limited or even conflicting, except that there was a moderate level of evidence for the association between BMLs and hip pain. This can be attributed to various reasons. Firstly, the limited number of included studies may have restricted the breadth and depth of the analysis, potentially leading to less robust conclusions. Secondly, some of the included studies might have exhibited lower overall quality of evidence due to factors such as small sample sizes and inadequate representativeness, impacting the reliability and validity of the findings. Moreover, our research methodology, which involved aggregating study results and applying uniform criteria, while simple, may have hindered the effective synthesis and interpretation of the data, potentially resulting in less accurate or comprehensive outcomes.

We found moderate evidence of a positive association between BMLs and hip pain. These findings are similar to other studies showing a significant association between BMLs and knee pain [11, 40], suggesting that BMLs could be a potential cause or indicator of both knee and hip OA. This could contribute to the management of hip OA, as effectively managing the progression of BMLs may reduce knee pain in knee OA with BMLs [18]. The additional MRI features in this study, despite showing limited or conflicting evidence, play a role in semi-quantitative evaluation of hip OA [41]. These features, awaiting further study, hold promise for distinguishing hip OA subtypes and informing its diagnosis and treatment.

The strength of this study is that we systematically screened studies that evaluated the association between hip MRI abnormalities and hip pain and employed a pre-specified assessment system to qualitatively evaluate the credibility of evidence. There are several limitations in this study. First, we categorized the results of the included studies as either negative or positive based solely on statistical significance, without considering the influence of sample size on the outcomes, and this may have overlooked false negative findings. However, the limited number of studies disabled us from conducting a meta-analysis to pool these results. Second, we scored the methodological quality of the included studies with different designs. The subjective awareness of the evaluator can have an impact on the results of the assessment, leading to biases, although the scores were rated by different authors to reach a consensus.

In conclusion, only a few studies with small to modest sample sizes evaluated the association between hip structural changes on MRI and hip pain. BMLs may contribute to the severity and progression of hip pain. Further studies are warranted to uncover the role of hip MRI abnormalities in hip pain.

## Electronic supplementary material

Below is the link to the electronic supplementary material.

Supplementary Material 1

Supplementary Material 2

Supplementary Material 3

## Data Availability

The data that support the findings of this study are available from the corresponding author upon reasonable request.
