# Supplementary material for: The relationship between MRI-detected hip abnormalities and hip pain in hip osteoarthritis: a systematic review
Source: Rheumatol Int. 2024 Aug 13;44(10):1887–96. doi: 10.1007/s00296-024-05678-2 (PMC11393093; doi:10.1007/s00296-024-05678-2)
Supplement: Supplementary file 3 — Supplementary Material 3 [file 296_2024_5678_MOESM3_ESM.docx]

Supplementary Table 1 The Newcastle-Ottawa Scale (NOS) for Assessing the Quality of Cross-sectional studies

| Studies | Kumar, 2013^[27]^ | Taljanovic, 2008^[28]^ | Kijima, 2020^[30]^ |
| --- | --- | --- | --- |
| A^^[[1]](#footnote-0)^^ | 1 | 0 | 0 |
| B^^[[2]](#footnote-1)^^ | 0 | 0 | 0 |
| C^^[[3]](#footnote-2)^^ | 1 | 1 | 1 |
| D^^[[4]](#footnote-3)^^ | 0 | 0 | 0 |
| E^^[[5]](#footnote-4)^^ | 2 | 1 | 1 |
| F^^[[6]](#footnote-5)^^ | 1 | 1 | 1 |
| G^^[[7]](#footnote-6)^^ | 1 | 1 | 1 |
| Total score | 6 | 4 | 4 |

Supplementary Table 2 The Newcastle-Ottawa Scale (NOS) for Assessing the Quality of Cohort Studies

| Studies | Ahedi, 2014[31] | Koyama, 2022[32] | Schwaiger, 2016[33] | Ahedi,  2016[34] | Ahedi, 2020[35] |
| --- | --- | --- | --- | --- | --- |
| A^[[8]](#footnote-7)^ | 1 | 0 | 1 | 1 | 1 |
| B^[[9]](#footnote-8)^ | 1 | 0 | 1 | 1 | 1 |
| C^[[10]](#footnote-9)^ | 1 | 1 | 1 | 1 | 1 |
| D^[[11]](#footnote-10)^ | 1 | 1 | 1 | 1 | 1 |
| E^[[12]](#footnote-11)^ | 2 | 2 | 2 | 2 | 2 |
| F^[[13]](#footnote-12)^ | 1 | 0 | 1 | 1 | 1 |
| G^[[14]](#footnote-13)^ | 1 | 1 | 0 | 1 | 1 |
| H^[[15]](#footnote-14)^ | 0 | 1 | 1 | 1 | 1 |
| Total score | 8 | 6 | 8 | 9 | 9 |

Supplementary 3 The Newcastle-Ottawa Scale (NOS) for Assessing the Quality of Case-control studies

| Studies | Heerey, 2021^[29]^ |
| --- | --- |
| A^^[[16]](#footnote-15)^^ | 1 |
| B^^[[17]](#footnote-16)^^ | 0 |
| C^^[[18]](#footnote-17)^^ | 1 |
| D^^[[19]](#footnote-18)^^ | 0 |
| E^^[[20]](#footnote-19)^^ | 2 |
| F^^[[21]](#footnote-20)^^ | 1 |
| G^^[[22]](#footnote-21)^^ | 1 |
| H^[[23]](#footnote-22)^ | 1 |
| Total score | 7 |

Supplementary Methods

We searched Medline (via Ovid) from inception to February 2024

1.exp osteoarthritis/

2.osteoarthr$.tw.

3.(degenerative adj2 arthritis).tw.

4.arthrosis.tw.

5.(1 or 2 or 3 or 4)

6.exp Magnetic Resonance Imaging/

7.((magnetic resonance or MR or NMR) adj2 (imag* or tomograph* or scan*)).tw.

8.(MRI or MRIs or NMRI).tw.

9.(6 or 7 or 8)

10.exp Pain/

11.hip/

12.hip$.tw.

13.exp hip joint/

14.(11 or 12 or 13)

15.(5 and 9 and 10 and 14)

16.limit 15 to humans

We searched Embase (via Ovid) from inception to February 2024

1.exp osteoarthritis/

2.osteoarthr$.tw.

3.(degenerative adj2 arthritis).tw.

4.arthrosis.tw.

5.(1 or 2 or 3 or 4)

6.exp Magnetic Resonance Imaging/

7.((magnetic resonance or MR or NMR) adj2 (imag* or tomograph* or scan*)).tw.

8.(MRI or MRIs or NMRI).tw.

9.(6 or 7 or 8)

10.exp Pain/

11.hip/

12.exp hip joint/

13.hip$.tw.

14.(11 or 12 or 13)

15.(5 and 9 and 10 and 14)

16.limit 15 to human

We searched Web of science from inception to February 2024

1.TS=(Osteoarthritis)

2.TS=(Degenerative Arthritis)

3.TS=(Arthrosis)

4.(1 or 2 or 3)

5.TS=(Magnetic Resonance Image)

6.TS=(MRI Scans)

7.(5 or 6)

8.TS=(pain)

9.TS=(hip)

10.TS=(hip joint)

11.(9 or 10)

12.(4 and 7 and 8 and 11)

We searched Cumulative Index to Nursing & Allied Health Literature (CINAHL) from inception to February 2024

1.MH=osteoarthritis

2.TX=osteoarthritis

3.TX=osteoarthrosis

4.TX=degenerative arthritis

5.TX=degenerative joint disease

6.TX=arthrosis

7.(1 or 2 or 3 or 4 or 5 or 6)

8.MH=magnetic resonance imaging

9.TX=magnetic resonance imaging

10.TX=magnetic resonance image

11.TX=nuclear magnetic resonance

12.TX=MRI scan

13.(8 or 9 or 10 or 11 or 12)

14.TX=pain

15.TX=hip

16.TX=hip joint

17.(7 and 13 and 14 and 15 and 16)

18.limit 17 to human

1. Representativeness of the sample [↑](#footnote-ref-0)
2. Sample size [↑](#footnote-ref-1)
3. Ascertainment of exposur [↑](#footnote-ref-2)
4. Non-respondents [↑](#footnote-ref-3)
5. The subjects in different outcome groups are comparable, based on the study design or analysis. Confounding factors are controlled [↑](#footnote-ref-4)
6. Assessment of outcome [↑](#footnote-ref-5)
7. Statistical test [↑](#footnote-ref-6)
8. Representativeness of the exposed cohort [↑](#footnote-ref-7)
9. Selection of the non-exposed cohort [↑](#footnote-ref-8)
10. Ascertainment of exposure [↑](#footnote-ref-9)
11. Demonstration that outcome of interest was not present at start of study [↑](#footnote-ref-10)
12. Comparability of cohorts on the basis of the design or analysis [↑](#footnote-ref-11)
13. Assessment of outcome [↑](#footnote-ref-12)
14. Was follow-up long enough for outcomes to occur [↑](#footnote-ref-13)
15. Adequate of follow-up of cohorts [↑](#footnote-ref-14)
16. Is the Case Definition Adequate? [↑](#footnote-ref-15)
17. Representativeness of the Cases [↑](#footnote-ref-16)
18. Selection of Controls [↑](#footnote-ref-17)
19. Definition of Controls [↑](#footnote-ref-18)
20. Comparability of Cases and Controls on the Basis of the Design or Analysis [↑](#footnote-ref-19)
21. Ascertainment of Exposure [↑](#footnote-ref-20)
22. Non-Response Rate [↑](#footnote-ref-21)
23. Same method of ascertainment for cases and controls [↑](#footnote-ref-22)
